# MicroRNA-155 Promotes Myocardial Infarction-Induced Apoptosis by Targeting RNA-Binding Protein QKI

**DOI:** 10.1155/2019/4579806

**Published:** 2019-05-05

**Authors:** Jing Guo, Hui-Bin Liu, Chuan Sun, Xiu-Qing Yan, Juan Hu, Jie Yu, Ye Yuan, Zhi-Min Du

**Affiliations:** ^1^Institute of Clinical Pharmacy, The Second Affiliated Hospital of Harbin Medical University (The University Key Laboratory of Drug Research, Heilongjiang Province), Harbin 150086, China; ^2^Department of Clinical Pharmarcology, College of Pharmacy, Harbin Medical University, Harbin 150086, China; ^3^State Key Laboratory of Quality Research in Chinese Medicines, Macau University of Science and Technology, Macau 999078, China

## Abstract

Acute myocardial infarction (AMI) is the leading cause of sudden death worldwide. MicroRNA-155 (miR-155) has been reported to target antiapoptotic genes in various diseases models, but the functional role of miR-155 in response to MI injury needs further investigations. This study investigated the role of miR-155 in myocardial ischemia injury. TUNEL and flow cytometry were performed to measure cell apoptosis. Western blot analysis was employed to detect protein expressions of Bcl-2, XIAP, Bax, and caspase-3. qRT-PCR was used to quantify miRNA levels. We showed that miR-155 was dynamically elevated in murine hearts subjected to MI and in neonatal rat ventricular cardiomyocyte (NRVM) injury induced by hydrogen peroxide (H_2_O_2_). In response to H_2_O_2_, the silencing of miR-155 using AMO-155 (antisense inhibitor oligodeoxyribonucleotides) significantly increased cell viability and reduced cell apoptosis. Moreover, AMO-155 reversed the H_2_O_2_-induced downregulation of Bcl-2 and XIAP and upregulation of Bax and cleaved-caspase-3. Further study revealed that AMO-155 resulted in a decrease of H_2_O_2_-induced JC-1-labelled monomeric cell number. In addition, AMO-155 markedly decreased infarct size, ameliorated impaired cardiac function, and significantly reduced apoptotic cell percentages in MI mice heart. The RNA-binding protein Quaking (QKI) was predicted as a target gene of miR-155 through bioinformatic analysis, and AMO-155 attenuated the downregulation of QKI in H_2_O_2_-treated cardiomyocytes and MI mice heart. Knockdown of QKI by siRNA abolished the antiapoptotic effects of AMO-155. Taken together, miR-155 is upregulated in the MI heart and NRVMs in response to H_2_O_2_ stress, and downregulating of miR-155 protects cardiomyocytes against apoptosis. Mechanistically, it is probably due to the repression of QKI signaling pathway.

## 1. Introduction

Acute myocardial infarction (AMI) can lead to left ventricular dilatation, heart failure, and sudden cardiac death, resulting in high morbidity and mortality worldwide [1]. Numerous experimental and clinical studies have shown that cardiomyocyte apoptosis occurs in the border zone close to myocardial infarcted area. It was usually caused by oxidative stress, ischemia, and hypoxic injury and reperfusion, subsequently aggravating cardiac dysfunction [2–5]. Thus, the inhibition of cardiomyocyte apoptosis during the initial stage of AMI is the key for repairing the injured heart and treating ischemic heart disease.

MicroRNAs (miRNAs) are a group of conserved, small, noncoding RNAs, typically 18~25 nucleotides in length, which manipulate target gene expressions [6]. Studies indicate that several miRNAs play crucial roles in cardiomyocyte apoptosis at present stage. Our previous study showed that miR-98 protected against MI-induced cardiomyocyte apoptosis and myocardial dysfunction [7]. In addition, miR-195 has the opposite effect in cardiomyocyte apoptosis [8]. Recent study elucidated that the loss function of miR-155 protects the heart from pathological cardiac hypertrophy [9]. Studies also suggested that miR-155 is involved in regulating cardiac fibrosis via the TGF-*β*1/Smad2 signaling pathway [10]. These findings proposed that miR-155 plays an important role in regulating cardiac disease. However, the role of miR-155 in cardiac ischemia-induced apoptosis remains unknown. The RNA-binding protein Quaking (QKI) has high expression in the adult heart and other organs [11, 12], which is involved in miRNA metabolic processing [13, 14]. The QKI gene encodes at least three protein isoforms (QKI-5, QKI-6, and QKI-7), and these isoforms have different carboxy-terminal ends [15]. At present, some evidences indicated that QKI has a vital function in the process of apoptosis. A study revealed that QKI could inhibit the ischemia/reperfusion-induced apoptosis in neonatal cardiomyocytes [16]. Another research also validated that QKI could depress doxorubicin-mediated cardiomyocyte apoptosis [17]. In addition, miR-155 was verified to target QKI directly in U937 cells [18]. In this study, we firstly investigated the changes of miR-155 expression in both cellular and mouse model of MI. We found that miR-155 was significantly upregulated in these two models, and the inhibition of miR-155 decreased apoptosis and preserved cell survival by the upregulation of QKI. Thus, the downregulation of miR-155 may provide a therapeutic target for MI in mice.

## 2. Materials and Methods

### 2.1. Animals

Healthy adult Kunming male mice weighing about 25-30 g were used in the current study. Before the experiment, these animals were kept under standard animal housing conditions in which temperature is 23 ± 1°C and humidity is 55 ± 5% and fed with food and water ad libitum for 1 week before the experiments. All procedures were performed in accordance with the Institutional Animal Care and Use Committee of the Harbin Medical University and the Ethic Committee of Harbin Medical University, China.

### 2.2. Neonatal Rat Ventricular Myocyte Culture and Transfection

Neonatal rat ventricular cardiomyocytes (NRVMs) were isolated from the ventricles of neonatal Sprague-Dawley rats (1-3 days old), which were obtained from the Experimental Animal Center of the Second Affiliated Hospital of Harbin Medical University, China. Briefly, the hearts of neonatal SD rats were sterilized in 75% ethanol and sacrificed by decapitation. Then hearts were isolated from the chest and minced in serum-free DMEM (HyClone, USA), and then each heart is separated into 6-8 pieces. Single ventricular myocyte was isolated by 0.25% trypsin solution. Pooled cell suspensions were centrifuged and then resuspended in DMEM containing 10% fetal bovine serum and 5% penicillin/streptomycin. Finally, the cells were plated into culture flasks (noncoated) and cultured under a condition of 5% CO_2_ and 95% air at 37°C for 2 hours. Two hours later, the fibroblasts were adhered under the microscope and then blew off cardiomyocytes lightly from culture flasks and plated into 96-well plates or 6-well plates. After two days, NRVMs were starved in serum-free medium for 24 hours and then transiently transfected with miR-155 (50 nM), AMO-155 (100 nM), NC (50 nM), or QKI siRNA (50 nM), using Opti-MEM®I (Invitrogen, USA) and X-treme GENE siRNA transfection reagent (Roche, Penzberg, Germany) according to manufacturer's protocols. After transfection for 44 hours, NRVMs were treated with 100 *μ*M hydrogen peroxide (H_2_O_2_) for 4 hours. The total time of transfection was 48 hours. The cells were then harvested for further studies. The miR-155 (5′-UUAAUGCUAAUUGUGAUAGGGGU-3′), anti-miR-155 antisense oligonucleotides (AMO-155) (5′-ACCCCUAUCACAAUUAGCAUUAA-3′), and QKI siRNA (siRNA1: 855-873: 5′-CATTAAATCACCAGCCCTT-3′; siRNA2: 1213-1231: 5′-GCTGGATGAAGGACTAGAA-3′; siRNA3:296-314: 5′-TGAACGACAAGAAGCTTAT-3′) were synthesized by Guangzhou Ribo Bio Co. Ltd. AMO-155 contained 2′-O-methyl modifications.

### 2.3. RNA Extraction and Real-Time PCR

Total RNA was extracted from cultured NRVMs or heart tissue using Trizol reagent (Invitrogen, USA) according to manufacturer's instructions. The expressions of miR-155 and QKI mRNA were detected using SYBR Green incorporation on Roche Light-Cycler 480 Real-Time PCR System (Roche, Germany), while U6 was used as an internal control for miR-155 and GAPDH for QKI. The sequences of primers used were listed as follows: miR-155 F: 5′-GCTTAATGCTAATTGTGATAGGGG-3′, R: 5′-CAGTGCGTGTCGTGGAGT-3′; U6 F: 5′-GCTTCGGCACATATACTAAAAT-3′, R: 5′-CGCTTCACGAATTTGCGTGTCAT-3′; QKI (rat) F: 5′-AGCGGTTGAAGAAGTGAAGAAG-3′, R: 5′-TTAATGTTGGCGTCTCTGTAGG-3′; and GAPDH (rat): F: 5′-GGAAAGCTGTGGCGTGAT-3′, R: 5′-AAGGTGGAAG AATGGGAGTT-3′. Quantitative real-time PCR was performed in 20 *μ*L volumes with SYBR Green PCR Master Mix (Roche, USA) at 95°C for 10 min and 40 cycles at 95°C for 15 s, 60°C for 30 s, and 72°C for 30 s, using Light Cycler 480 (Roche, USA). The amount of target (2^-ΔΔCT^) was calculated by normalizing to endogenous reference and relative to an average of the control samples, calibrator.

### 2.4. MTT Assay

The NRVMs were seeded in 96-well culture plates, and the number of adherent cells reached 2 × 10^4^ cells per well. Cell viability was tested by 3-(4,5-dimethylthiazol-2-yl)-2,5-diphenyltetrazolium bromide (MTT) assay according to manufacturer's protocols. The absorbance was calculated at 490 nm by Microplate Reader (Infinite M200, TECAN).

### 2.5. TUNEL Assay

Apoptosis of cardiomyocytes was detected by staining ventricular specimens from border zone (3 days post-MI) and neonatal rat cardiomyocytes (4 hours after 100 *μ*M H_2_O_2_) with the *In Situ* Cell Death Detection Kit (TUNEL fluorescence FITC kit, Roche) according to manufacturer's protocols. After TUNEL staining, the NRVMs or ventricular specimens were immerged into the DAPI (Sigma-Aldrich) solution to stain the nuclei of living and apoptotic cells. Laser scanning confocal microscope (Olympus, Fluoview1000, Tokyo, Japan) was used to view the fluorescence staining.

### 2.6. Annexin V-FITC/Propidium Iodide (AV/PI) Dual Staining

The Annexin V-FITC/Propidium Iodide (AV/PI) Apoptosis Detection Kit (Vazyme, Nanjing, China) was utilized to determine necrosis (Annexin V-FITC-/PI+, Q1), early apoptosis (Annexin V-FITC+/PI-, Q4), and late apoptosis (Annexin V-FITC+/PI+, Q2), according to manufacturer's protocol (Vazyme, Nanjing, China). As our previous study reported [7], the adherent cardiomyocytes were digested with 0.25% trypsin, washed by phosphate buffer solution (PBS), dual-stained with AV and PI, and then analyzed by flow cytometry (BD Bioscience, USA).

### 2.7. Western Blot Analysis

The concentration of total proteins from different regions of the left ventricular myocardium or NRVMs was determined with BCA Protein Assay Kit (Beyotime, Shanghai, China). Proteins were separated by electrophoresis on SDS-PAGE (10% or 12.5% polyacrylamide gels) and then transferred to nitrocellulose membrane. Subsequently, nitrocellulose membranes were blocked in 5% nonfat milk PBS for 2 hours and then incubated overnight at 4°C with anti-Bax (1 : 1000, Proteintech, USA), anti-Bcl-2 (1 : 1000, Cell Signaling Technology, USA), anti-caspase-3 (1 : 1000, Cell Signaling Technology, USA), anti-QKI (1 : 1000, 13169-1-AP, Proteintech, USA), or *β*-actin (1 : 1000, ZSGB-Bio, China) primary antibodies, followed by incubation for 1 hour at room temperature with IRDye secondary antibodies (LI-COR). The Odyssey CLx Infrared Imaging System (LI-COR Biosciences, Lincoln, NE, USA) was used to capture the images, and Odyssey CLx version 2.1 was used to quantify western blot bands by measuring the intensity in each group. The data was normalized to *β*-actin as an internal control.

### 2.8. MI Model and Administration of AntagomiR-155

Adult male Kunming mice (25-30 g) were randomly divided into three groups: sham, MI, and MI+antagomiR-155, respectively. The antagomiR-155 (Ribo Bio, Guangzhou, China) is identical to the mature mmu-miR-155-5p (5′-UUAAUGCUAAUUGUGAUAGGGGU-3′) which is single-stranded RNA analogues with chemically modified and conjugated with cholesterol moiety for *in vivo* applications with long-lasting stability and enhanced target specificity and affinity. The animals were anesthetized with 2,2,2-Tribromoethanol (Sigma, USA) via i.p. (20 mg/kg) and were orally intubated with 20-gauge tube and ventilated (mouse ventilator, PhysioSuite, Kent Scientific Corporation, USA) at a respiratory rate of 120 breaths/min and a tidal volume of 1.50 mL. The standard limb lead ECG was continuously recorded on a recorder (BL-420, Taimeng, Chengdu, China). A left thoracotomy was performed between the 3rd rib and the 4th rib and the heart was exposed. AntagomiR-155 was injected into the left ventricular cavity through the tip of the heart at a dosage of 200 nmol/kg in 0.08 mL saline, and the aortic artery and main pulmonary artery were clamped for 10 seconds using a bulldog clamp. An equal volume of saline was given for sham mice. Then, a segment of saline-soaked 8-0 sutures was then looped around the left anterior descending (LAD) coronary artery to induce the infarction of the left ventricular free wall. Cardiac infarction was confirmed by cyanosis of the myocardium and apparent S-T segment elevation in ECG. The chest cavity was then sutured by a segment of saline-soaked 5-0 sutures and the thorax closed. Sham-operated mice underwent the same surgical procedure but without tying the thread.

### 2.9. Echocardiographic Measurements

Three days after MI, the cardiac function of mice was determined by transthoracic echocardiography with an ultrasound machine (Panoview *β*1500, Cold Spring Biotech, Taiwan, China) equipped with a 30 MHz phased-array transducer as described previously [7]. M-mode tracings were used to detect percentage of left ventricular ejection fraction (EF%) and fractional shortening (FS%).

### 2.10. Measurement of Infarct Size

Three days after MI, mice were euthanized under deep anesthesia by intraperitoneal injection of Avertin (2,2,2-Tribromoethanol) (Sigma-Aldrich) (20 mg/kg). The whole heart was cut into 2 mm thick slices and stained with 1% triphenyltetrazolium chloride (TTC) at 37°C for 20 minutes after washing out remaining blood and the infarct area (IA) then was stainless while the live area turned red. Then, the infarct size was determined from the weight ratio of IA/LV (IA: infarct area, LV: left ventricles). For further study, the tissues in the ischemic area of the hearts were collected and stored at −80°C.

### 2.11. LDH Activity Assay

Serum LDH activity was determined by colorimetric assay kits (Nanjing Jiancheng Bioengineering Institute, Nanjing, China) as described in our previous study [7]. Three days after MI, the blood samples of mice were collected and the activity of LDH was assessed with the colorimetric method according to manufacturer's instruction.

### 2.12. Luciferase Reporter Assay

To construct the QKI expression plasmids, the wide-type or mutant 3'UTR of QKI gene was cloned into the pmiR-RB-Report vector. For luciferase assays, HEK293T cells were seeded in a 96-well plate and cotransfected with 100 ng plasmid and miR-155 mimics or negative controls using Lipofectamine 2000 reagent according to manufacturer's instructions. The cells were collected after 48 hours and firefly and Renilla luciferase activities were measured by Dual-Luciferase Reporter Assay System (Promega, Madison, WI, USA).

### 2.13. Data Analysis

All data were presented as mean ± SEM and analyzed by SigmaPlot and SigmaStat Software (Jandel Scientific, CA, USA). Student's *t*-test or ANOVA (followed by Student-Newman-Keuls post hoc test) was used where appropriate. Differences were considered statistically significant for *P* < 0.05.

## 3. Results

### 3.1. miR-155 Is Upregulated in Ischemic Heart and H_2_O_2_-Treated Cardiomyocytes

**Figure 1:**
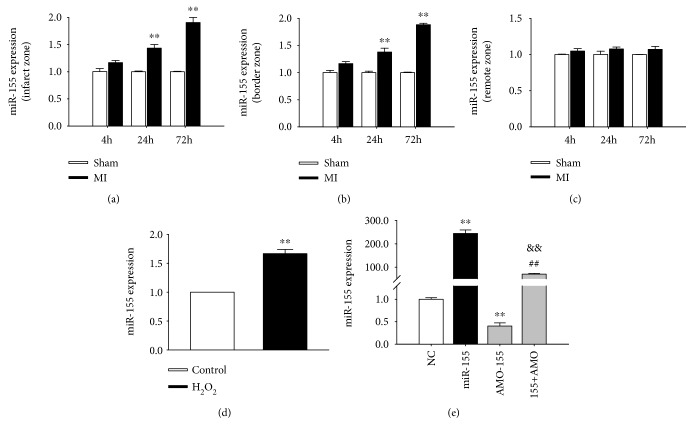
miR-155 upregulated in MI mice and H_2_O_2_-treated NRVMs. (a, b, c) miR-155 expression in the infarcted, border, and remote zones 24 h and 72 h after myocardial infarction (MI) in mice was detected by qRT-PCR. *n* = 3; ^∗∗^*P* < 0.01 vs. sham. (d) miR-155 expression in H_2_O_2_-treated NRVMs was detected by qRT-PCR. *n* = 6; ^∗∗^*P* < 0.01 vs. control. (e) miR-155 level was detected by qRT-PCR after treatment with miR-155 mimics or AMO-155 transfection in NRVMs. *n* = 6; ^∗∗^*P* < 0.01 vs. NC; ^##^*P* < 0.01 vs. +miR-155; ^&&^*P* < 0.01 vs. AMO-155.

We first examined miR-155 expression in infarcted zone, border zone, and remote zone after MI for 4 h, 24 h, and 72 h, respectively. qRT-PCR data suggested that miR-155 was much more expressed in the infarcted zone of MI mice hearts at 24 h and 72 h than that in sham-operated animals (Figure 1(a)). Additionally, in the border zone of MI mice hearts at 24 h and 72 h, miR-155 was much higher than that in sham-operated animals (Figure 1(b)). In contrast, no change of miR-155 was observed in the remote zone of MI mice (Figure 1(c)). Meanwhile, in line with the results shown above, miR-155 expression was also increased by 66% in NRVMs after 100 *μ*M H_2_O_2_ treatment (Figure 1(d)).

Then, miR-155 or AMO-155 was transfected into NRVMs to overexpress or knockdown miR-155 expression. The qRT-PCR results showed that miR-155 level was increased by about 244% after transfecting with mimics-155 (Figure 1(e)). Meanwhile, AMO-155 transfection lead to a 40% decreased expression of miR-155 in NRVMs, which was rescued by coapplication of both mimics and AMO-155 (Figure 1(e)). Above data suggested that the overexpression or knockdown of miR-155 was successfully transfected in the cardiomyocytes.

### 3.2. AMO-155 Prevents Cardiomyocyte Apoptosis in Response to H_2_O_2_

**Figure 2:**
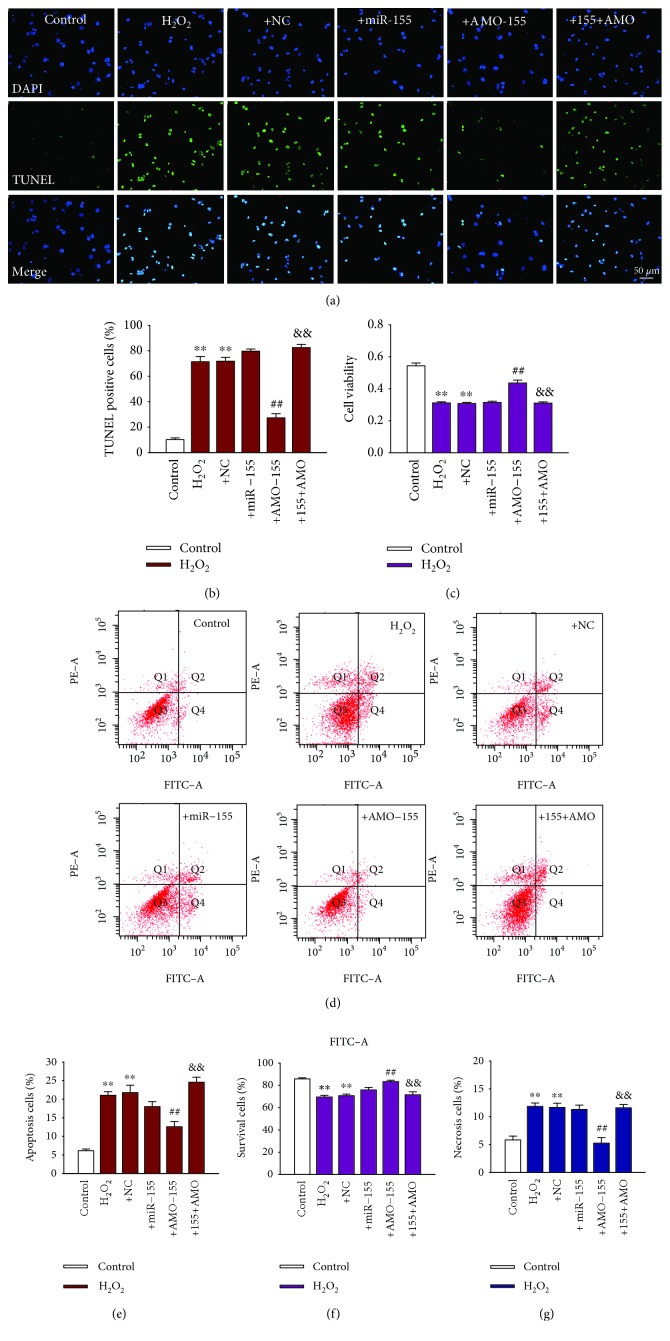
AMO-155 attenuated cardiomyocyte apoptosis in response to H_2_O_2_. NRVMs were transfected with NC, miR-155, AMO-155, and miR-155+AMO-155 (155+AMO) and then treated with H_2_O_2_ (100 *μ*M) for 4 h. (a) Representative images of TUNEL staining of NRVMs for DNA defragmentation showing the apoptotic cells (nucleus stained in blue with DAPI and apoptotic cells stained in green). (b) Quantification analysis of total TUNEL-positive cells. *n* = 6. (c) The cell viability of NRVMs treated with 100 *μ*M H_2_O_2_ for 4 h detected by MTT assay. *n* = 8. (d) The representative images of flow cytometry using double staining of Annexin V and PI. (e–g) Statistical analysis of apoptotic, survival, and necrosis ratio of the flow cytometry data. *n* = 4; ^∗∗^*P* < 0.01 vs. control; ^##^*P* < 0.01 vs. +NC; ^&&^*P* < 0.01 vs. +AMO-155. +NC: H_2_O_2_+NC; +155: H_2_O_2_+miR-155; +AMO-155: H_2_O_2_+AMO-155; +155+AMO: H_2_O_2_+miR-155+AMO-155.

We then evaluated the role of AMO-155 on cell apoptosis. It was evaluated by TUNEL staining. TUNEL-positive cells increased by 60% after treatment with 100 *μ*M H_2_O_2_ for 4 h on NRVMs. Silencing of miR-155 by AMO-155 (100 nmol/L), the specific inhibitor of miR-155, inhibited H_2_O_2_-induced apoptosis of NRVMs, whereas cotransfection with miR-155 (50 nmol/L) abrogated the effects of AMO-155 (Figures 2(a) and 2(b)). Furthermore, H_2_O_2_ treatments showed decreased by about 40% cell viability by MTT assay (Figure 2(c)). Transfection with AMO-155 (100 nmol/L) suppressed H_2_O_2_-induced impaired cell viability, which was abolished by cotransfection of miR-155 (Figure 2(c)). In addition, flow cytometry was utilized to investigate the protective role of AMO-155 in H_2_O_2_-induced cell apoptosis. Our data showed that the percentage of apoptotic cells was raised by about 3.5 times in H_2_O_2_-treated cells, which was downregulated by 40% by AMO-155 (Figures 2(d) and 2(e)). Similarly, the antiapoptotic effects of AMO-155 were abolished by cotransfection with miR-155 (Figures 2(d) and 2(e)). Moreover, AMO-155 also elevated cell viability and diminished cell necrosis in H_2_O_2_-treated NRVMs, which were rescued by the coapplication of miR-155 to overexpress miR-155 (Figures 2(f) and 2(g)). Collectively, these results suggested that decreased miR-155 expression protected cardiomyocyte against H_2_O_2_ stimuli and promoted cardiomyocyte survival.

### 3.3. Effect of miR-155 on Apoptosis-Related Protein Expression and Mitochondrial Membrane Potential (Δ*ψ*m)

**Figure 3:**
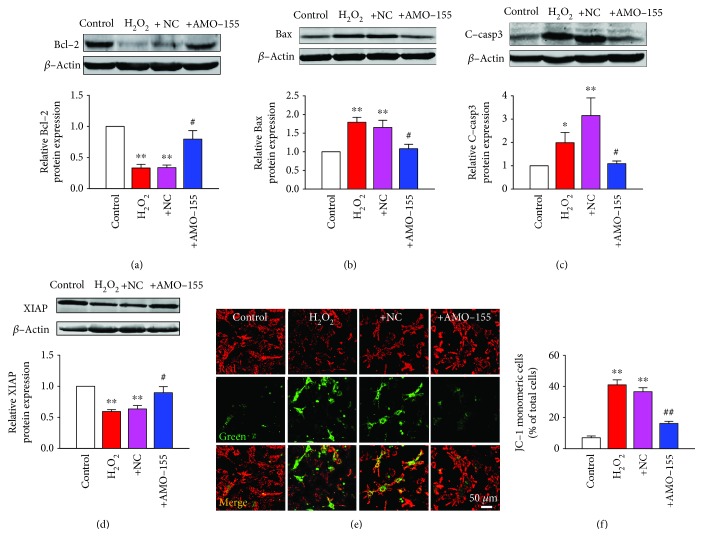
Effect of miR-155 on apoptosis-related protein expression and mitochondrial membrane potential (Δ*ψ*m). (a) Western blot analysis of relative Bcl-2 protein expression in NRVMs. *n* = 4. (b) Western blot analysis of relative Bax protein expression in NRVMs. *n* = 4. (c) Western blot analysis of relative C-casp3 protein expression in NRVMs. *n* = 6. (d) XIAP expression level was detected by western blot. *n* = 4. (e) Representative fluorescent images of JC-1 monomeric mitochondria showing green fluorescence and JC-1-aggregated mitochondria showing red from control, H_2_O_2_, H_2_O_2_+NC, and H_2_O_2_+AMO-155 groups. (f) Statistical analysis of mitochondrial membrane potential labelled by JC-1 monomeric. *n* = 5. ^∗^*P* < 0.05, ^∗∗^*P* < 0.01 vs. control; ^#^*P* < 0.05, ^##^*P* < 0.01 vs. +NC. +NC: H_2_O_2_+NC; +AMO-155: H_2_O_2_+AMO-155; C-casp3: cleaved-caspase-3.

Since AMO-155 prevented cardiomyocyte apoptosis and promoted cell survival, we further assessed its effect on regulating mitochondrion pathway. Our data showed that the exposure of NRVMs to H_2_O_2_ induced a lower expression level of Bcl-2, which was reversed by the downregulation of miR-155 (Figure 3(a)). In addition, as shown in Figure 3(b), H_2_O_2_ exposure increased by 1.7 times the expression of Bax, a proapoptotic protein. Nevertheless, AMO-155 alleviated the elevation of Bax expression induced by H_2_O_2_ in NRVMs compared with the NC group (Figure 3(b)). Cleaved-caspase-3 (C-casp3), the activation form of caspase-3, increased in NRVMs after exposure to H_2_O_2_ but decreased after AMO-155 transfection (Figure 3(c)). A prosurvival protein XIAP (X-chromosome-linked inhibitor of apoptosis protein) was downregulated in NRVMs treated with H_2_O_2_ but significantly upregulated by 20% by AMO-155 (Figure 3(d)). Furthermore, we assessed the role of AMO-155 in regulating mitochondrial membrane potential (Δ*ψ*m) assessed as changes in JC-1 fluorescence. The increase in the number of JC-1 monomeric cells (green) indicated the loss of Δ*ψ*m. As shown in Figures 3(e) and 3(f), the application of H_2_O_2_ increased JC-1 signal and the response could be attenuated with AMO-155 transfection.

### 3.4. Reduction of Infarct Size and Improvement of Cardiac Function by Antagomir-155 in MI Mice

**Figure 4:**
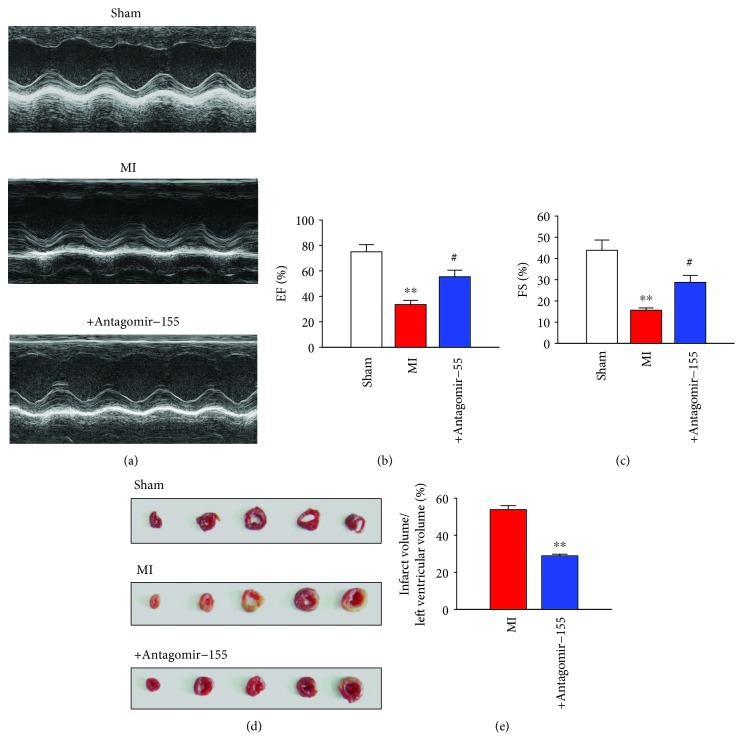
Reduction of infarct size and improvement of cardiac function by AMO-155 in MI mice. (a) Representative photographs of heart function detected by echocardiography examination. Statistical analysis of percentages of (b) ejection fractions (EF) and (c) fractional shortening (FS) from echocardiography examination. *n* = 6. ^∗∗^*P* < 0.01 vs. sham group; ^#^*P* < 0.05 vs. MI group. (d) Representative images of infarct areas in cross section slices of mice heart. (e) Statistical analysis of IA/LV ratio. IA: infarct area; LV: left ventricles. *n* = 3. ^∗∗^*P* < 0.01 vs. MI group.

Before coronary artery ligation, antagomir-155 was administered and then the effect of miR-155 inhibition in the infarcted heart was investigated. Cardiac function detected by echocardiography examination showed decreased percentages of ejection fraction (EF) and fractional shortening (FS) by 56% and 66%, respectively, in MI hearts (Figures 4(a)–4(c)). The inhibition of miR-155 by antagomir-155 significantly alleviated the impairment of left ventricular performance, as indicated by the increased EF% and FS% (Figures 4(a)–4(c)). Additionally, antagomir-155 reduced the infarct size by about 46% in MI mice heart (Figures 4(d) and 4(e)).

### 3.5. Antagomir-155 Protected Cardiomyocytes against Ischemia-Induced Apoptosis in a Mouse MI Model

**Figure 5:**
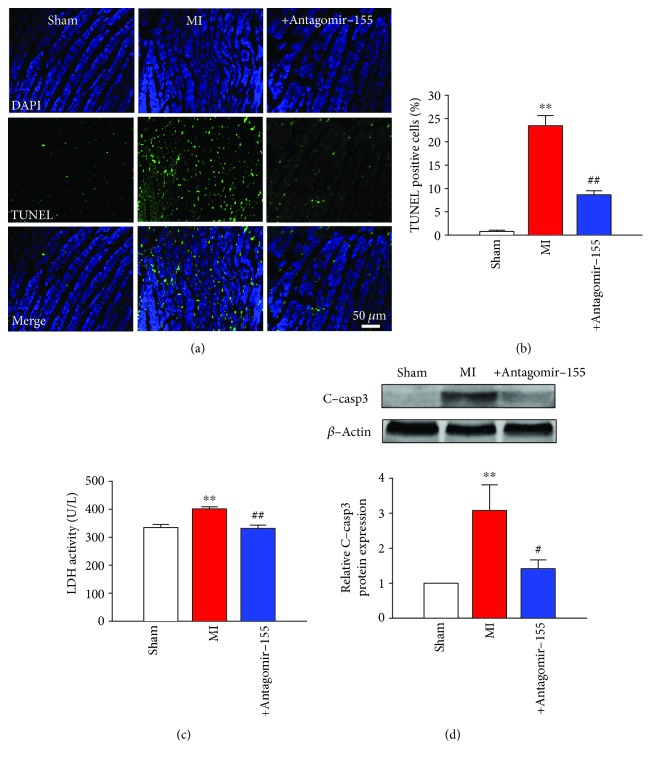
Antagomir-155 protected cardiomyocytes against ischemia-induced apoptosis in a mouse MI model. (a) Representative images of TUNEL staining of cells from ventricular sections for DNA defragmentation showing the apoptotic cells (nucleus stained in blue with DAPI and apoptotic cells stained in green); *n* = 6. (b) Quantification analysis of total TUNEL-positive cells from different groups. (c) Serum lactate dehydrogenase (LDH) activity of different groups detected by LDH kit; *n* = 6. (d) C-casp3 protein expression in MI mice was detected by western blot; *n* = 5. ^∗∗^*P* < 0.01 vs. sham group; ^#^*P* < 0.05, ^##^*P* < 0.01 vs. MI group. C-casp3: cleaved-caspase-3.

We then tried to clarify whether the antiapoptotic effects of antagomir-155 in vivo conditions in MI were in accord with AMO-155 on cultured cells under H_2_O_2_ conditions. Figures 5(a) and 5(b)illustrated that cardiomyocyte apoptosis significantly increased after MI in mice. The TUNEL-positive cells in the MI group increased by about 2 times. Nevertheless, this ischemic apoptosis drastically decreased by 55% after treatment with antagomir-155. In addition, the activity of serum lactate dehydrogenase (LDH), a marker for cardiac injury, elevated by about 19% after MI, while antagomir-155 administration obviously decreased this elevation by 19% (Figure 5(c)). Furthermore, we test the alteration of caspase-3 activity in response to MI stimuli. As shown in Figure 5(d), the expression level of cleaved-caspase-3 (C-casp3) was tripled in MI mice heart as compared to sham-operated mice heart. In contrast, the elevation of C-casp3 expression induced by MI was decreased by 53% by antagomir-155 administration (Figure 5(d)).

### 3.6. QKI as a Target Gene for miR-155 in Cardiomyocytes

**Figure 6:**
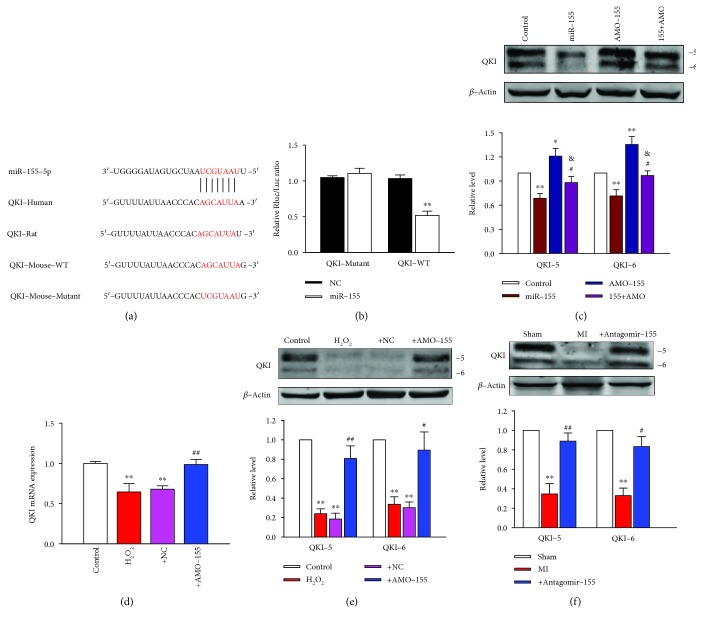
QKI as a target gene for miR-155 in cardiomyocytes. (a) The bioinformatic analysis showed that miR-155 had a binding site in the 3'-UTR of QKI mRNA of rat, human, and mouse species. (b) Relative luciferase activities of QKI wild-type (QKI-WT) UTRs and mutant UTRs of QKI (QKI-Mutant) were obtained by cotransfection of negative control miRNA or miR-155 mimics and the recombinant plasmid. Relative luciferase activity was calculated as the ratio of Renilla/firefly activities in the cells and normalized to those of the control; *n* = 8. ^∗∗^*P* < 0.01 vs. NC. (c) The protein levels of QKI-5 and QKI-6 in NRVMs were tested by western blot; *n* = 5. ^∗^*P* < 0.05, ^∗∗^*P* < 0.01 vs. control; ^#^*P* < 0.05 vs. miR-155; ^&^*P* < 0.05 vs. AMO-155. (d) QKI mRNA level was detected by qRT-PCR; *n* = 4. ^∗∗^*P* < 0.01 vs. control; ^##^*P* < 0.01 vs. +NC. (e) The protein expression of QKI-5 and QKI-6 in NRVMs were tested by western blot; *n* = 4. ^∗∗^*P* < 0.01 vs. control; ^#^*P* < 0.05, ^##^*P* < 0.01 vs. +NC. (f) The protein expression of QKI-5 and QKI-6 in mice were tested by western blot; *n* = 3. ^∗∗^*P* < 0.01 vs. sham group; ^#^*P* < 0.05, ^##^*P* < 0.01 vs. MI group.

QKI links intracellular signaling to cellular survival and QKI dysregulation is implicated in several cardiovascular diseases. As illustrated in Figure 6(a), the 3'UTR of QKI has one binding site of miR-155 in humans, mice, and rats. Previous study has experimentally identified QKI as a target gene of miR-155 in humans by luciferase assay [18]. In this study, we further confirmed the direct regulation of QKI by miR-155 using luciferase assay in our study. Luciferase reporters containing the 3'UTR fragment of QKI encompassing the miR-155 binding sites (QKI-WT) or a mutated fragment (QKI-Mutant) were constructed and transfected into HEK293T cells. We found that miR-155 overexpression only decreases the luciferase activity in wild-type reporter but not mutant one (Figure 6(b)), suggesting that miR-155 represses QKI by physically binding to the 3'UTR of this gene.

We then tested the influence of miR-155 on the expression of QKI in NRVMs. There was evidence that the QKI protein has two isoforms [16, 19]. The overexpression of miR-155 inhibits QKI protein levels in NRVMs, which was alleviated by cotransfection of AMO-155 (Figure 6(c)). In contrast, AMO-155 enhances the expression of QKI proteins and the action of AMO-155 was counteracted by cotransfection of miR-155 (Figure 6(c)), indicating the specificity of miR-155 action. Furthermore, as shown in Figure 6(d), QKI mRNA expression was downregulated 36% in the H_2_O_2_-treated NRVMs compared with the control group, which could be alleviated by AMO-155. Moreover, it is noticeable that the protein level of QKI-5 and QKI-6 was decreased 77% and 67%, respectively, in H_2_O_2_-treated NRVMs compared with the control group. Inhibition of miR-155 expression led to an increased QKI-5 and QKI-6 protein expression in posttranscriptional level, further indicating that QKI was the target gene of miR-155 (Figure 6(e)). We also examined the protein expression of QKI-5 and QKI-6 in MI mouse. The protein expression of QKI-5 and QKI-6 decreased by 65% and 67%, respectively, in MI mice, compared with sham mice, and it was reversed by miR-155 antagomir administration (Figure 6(f)).

### 3.7. QKI Inhibition Abolishes Protective Effect of AMO-155 on Cardiomyocytes

**Figure 7:**
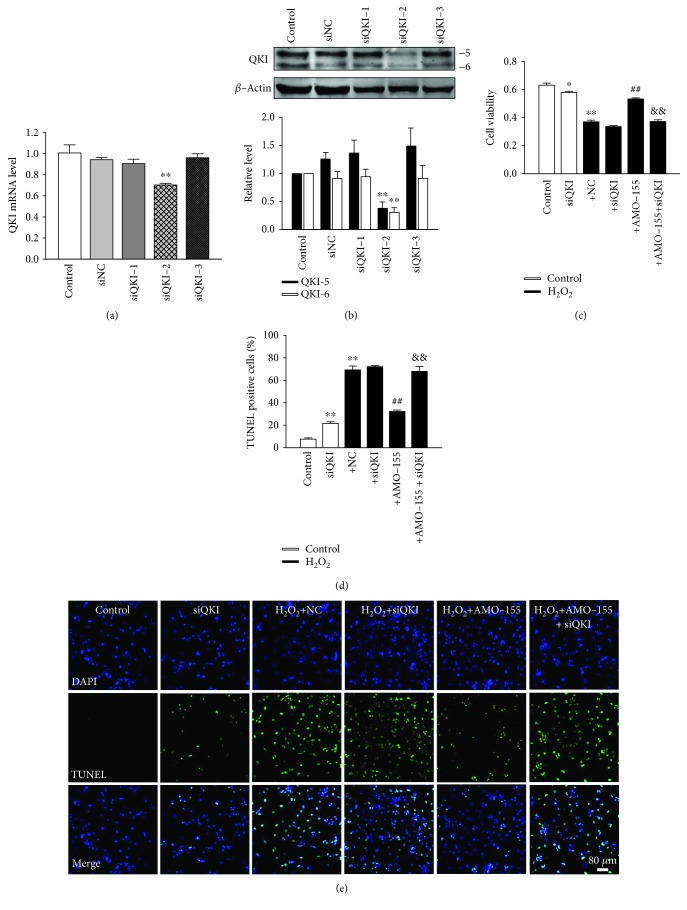
QKI inhibition abolishes protective effect of AMO-155 on cardiomyocytes. (a, b) QKI was silenced through siRNA transfection, and qRT-PCR and western blot were performed to detect the mRNA and protein level of QKI. siQKI-2 was used to downregulate QKI in the following experiments. (c) The cell viability of NRVMs after treatment with silencing QKI detected by MTT assay; *n* = 6. (d) Statistical results of TUNEL-positive cells per field. (e) Representative images of TUNEL staining of NRVMs showing the apoptotic cells; *n* = 4. ^∗^*P* < 0.05. ^∗∗^*P* < 0.01 vs. control; ^##^*P* < 0.01 vs. +NC; ^&&^*P* < 0.01 vs. +AMO-155. +NC: H_2_O_2_+NC; +siQKI: H_2_O_2_+siQKI; +AMO-155: H_2_O_2_+AMO-155; +AMO-155+siQKI: H_2_O_2_+AMO-155+siQKI.

Then we explored whether QKI was directly involved in the regulation of miR-155 on H_2_O_2_-induced cardiomyocyte apoptosis. We used siRNA technology to directly silence QKI gene in NRVMs. The qRT-PCR results showed that endogenous QKI mRNA was significantly reduced in NRVMs transfected with siQKI-2, as compared to the cardiomyocytes transfected with the siRNA-negative control (siNC) and other two QKI siRNAs (Figure 7(a)). The effect of siQKI-2-silencing QKI gene also was verified by western blot. As shown in Figure 7(b), siQKI-2 dramatically decreased QKI-5 and QKI-6 protein expression. Based on the above results, we chose siQKI-2 in subsequent experiments. As shown in Figure 7(c), AMO-155-mediated elevation of cell viability of H_2_O_2_-treated NRVCs was inhibited by siQKI. In addition, the transfection of AMO-155 markedly suppressed 54% of cardiomyocyte apoptosis and this effect was abolished by siQKI (Figure 7(d)). Representative TUNEL-stained photomicrographs from cardiac myocytes with different treatment are displayed in Figure 7(e). Therefore, these data suggested that the downregulation of QKI is involved in miR-155-mediated apoptosis in cardiomyocytes.

## 4. Discussion

In this study, we demonstrated the proapoptotic property of miR-155 in MI model and clarified the molecular mechanism underlying the protective effect of miR-155 inhibition in MI. Our data showed that miR-155 was dramatically increased in MI mice and that the knockdown of miR-155 prevented cardiomyocyte apoptosis and improved heart function. Furthermore, QKI was identified as a target of miR-155, and the knockdown of QKI promoted cardiac apoptosis and alleviated the antiapoptotic effect of miR-155 inhibition. These new findings allowed us to conclude that miR-155 is a novel proapoptotic miRNA, and the inhibition of miR-155 could be an effective therapeutic approach to prevent or minimize myocardial infarction.

Several previous studies illustrate that miR-155 participates in the regulation of heart diseases. It has been shown that the inhibition of miR-155 expression in cardiac fibroblasts can improve myocardial remodeling by targeting TP53INP1 [20]. In addition, miR-155 also participates in regulating migration of human cardiomyocyte progenitor cells by targeting MMP-16 [21]. Furthermore, the knockdown of miR-155 improved myocardial injury and dysfunction induced by viral myocarditis via regulating macrophage polarization [22]. Our results demonstrated that the knockdown of miR-155 inhibited H_2_O_2_-induced apoptotic cell death via intrinsic pathway, also called “mitochondrion pathway.” In addition, the inhibition of miR-155 with its antagomir attenuates cardiomyocyte apoptosis and myocardial infarction sizes in MI heart. On the basis of our research, a growing number of studies have shown that miR-155 played a crucial role in regulating the apoptosis process. An earlier study suggested that miR-155 inhibitors significantly decreased the apoptosis rate on BV2 cell, and the inhibition of miR-155 may play protective roles in ischemic stroke [23]. In addition, blocking of miR-155 by its antagomir prevented cardiac apoptosis stimulated by LPS in mice [22]. The above researches and our data investigated that depressing miR-155 expression may play a protective role in apoptosis progress, but there were also studies showing that miR-155 had the opposite effect. de Santis et al. reported that miR-155 directly targeted and downregulated caspase-3 mRNA expression to prevent apoptosis of macrophages [24]. Another report revealed that miR-155 inhibited the apoptosis of THP-1 cells induced by *Mycobacterium tuberculosis* via directly targeted FoxO3 [25]. In addition, miR-155 was shown to attenuate the macrophage apoptosis by targeting FADD and forced expression of FADD blocked the antiapoptotic action of miR-155 [26]. Given the fact that the activity and response of miRNA vary depending on their upstream activators and the expression level of miRNA also differs in various cell types, we therefore speculated that these different effects of miR-155 on apoptosis may at least partly be explained by distinct species and animal models.Further detailed studies will be necessary to clarify this variation of miR-155-dependent activity of apoptosis in different cell types.

Quaking (QKI) is a RNA-binding protein, belonging to the signal transduction and activator of RNA (STAR) family [27], which posttranscriptionally regulates pre-mRNA splicing, mRNA turnover, mRNA stability, translation efficiency, or RNA transportation [28–30]. QKI links intracellular signaling to cellular survival and QKI dysregulation is implicated in several cardiovascular diseases. For example, QKI expression was deficient in diabetic hearts, which contributed to the overactivation of FoxO1 and subsequently enhances the ischemic intolerance of diabetic hearts [31]. In addition, knocking down endogenous QKI enhanced cardiomyocyte susceptibility to apoptotic stimuli, whereas overexpression of QKI suppressed ischemia/reperfusion-induced apoptosis in cardiomyocytes [16]. Furthermore, LPS-stimulated macrophage-induced electrical remodeling in atrial fibrillation was associated with reduced QKI expression [32]. More recently, it has been shown that overexpression of QKI obviously attenuates doxorubicin-induced cardiotoxicity via regulating a set of circular RNAs [17]. Consistent with the above findings, our data demonstrate dramatic downregulation of QKI in MI heart tissues and oxidative stimuli treated cardiomyocytes. Downregulating QKI expression, especially the QKI-5 and QKI-6 isoforms, also induced cardiomyocyte apoptosis in vitro. Furthermore, the inhibitory effect of miR-155 on NRVM apoptosis was reversed by the downregulation of QKI expression, demonstrating the important contribution of QKI in cardiac protection triggered by miR-155 silencing. In the current study, we also found that there is one miR-155 target site in QKI-3'-UTR which is highly conserved across vertebrates (Figure 6(a)). Moreover, evidence was shown that QKI was directly targeted by miR-155 in macrophages and colon cells and the direct binding site had been verified by luciferase report assay [18, 33]. Most importantly, in this study, we found that the levels of QKI-5 and QKI-6 were inversely correlated with miR-155 expression in cardiomyocytes (Figure 6). The abovementioned facts indicated that miR-155 might execute its proapoptotic function at least partly by targeting QKI. Since the main functions of QKI are regulating RNA splicing, stability, transportation, and protein translation, the exact mechanisms involving QKI-mediated antiapoptotic effect and its downstream regulation pathway require further investigation.

Previous studies have found that the key to QKI's antiapoptotic effect is through interaction with FoxO1 which is a transcription factor regulating cell death, cell growth inhibition, and glucose utilization [34]. QKI could inhibit ischemia/reperfusion-induced cardiomyocyte apoptosis, and it was achieved by downregulating and inactivating downstream proapoptotic transcription factor FoxO1 either directly or indirectly. Another study also provided evidence of regulation FoxO1 by QKI-5 [16]. The upregulation of QKI-5 reduced the expression of FoxO1 and the stress of NS and ER in ob/ob myocardium and further reduced the injury of myocardial infarction/reperfusion. QKI-5 deficiency led to the overactivation of FoxO1 in ob/ob animals, which then intensifies nitrosative stress and ER stress, and enhanced ischemic intolerance in diabetic hearts [31]. Based on the above evidence, QKI could inhibit cell apoptosis via depressing proapoptotic transcription factor FoxO1.

## 5. Conclusions

In summary, the present study elucidates that miR-155 inhibition represses the cardiomyocyte apoptosis, improves the cardiac function, and reduces the MI size by targeting QKI. Our study suggested that miR-155 inhibition-mediated upregulation of QKI may provide novel idea for the treatment of ischemic heart diseases.

## Data Availability

The data used to support the findings of this study are available from the corresponding author upon request.
